# Genotoxicity Associated with ^131^I and ^99m^Tc Exposure in Nuclear Medicine Staff: A Physical and Biological Monitoring Study

**DOI:** 10.3390/cells11101655

**Published:** 2022-05-16

**Authors:** Justyna Miszczyk, Aleksander Gałaś, Agnieszka Panek, Aldona Kowalska, Magdalena Kostkiewicz, Eliza Borkowska, Kamil Brudecki

**Affiliations:** 1 Department of Experimental Physics of Complex Systems, Institute of Nuclear Physics, Polish Academy of Sciences, 31-342 Kraków, Poland; agnieszka.panek@ifj.edu.pl; 2 Chair of Epidemiology and Preventive Medicine, Department of Epidemiology, Jagiellonian University Medical College, 31-008 Kraków, Poland; aleksander.galas@uj.edu.pl; 3 Department of Endocrinology and Nuclear Medicine, Holy Cross Cancer Center, 25-734 Kielce, Poland; aldona.kowalska@onkol.kielce.pl; 4 Faculty of Health Sciences, Jan Kochanowski University, 25-369 Kielce, Poland; 5 Heart and Vascular Diseases Department, Faculty of Medicine, Institute of Cardiology, Collegium Medicum, Jagiellonian University, 31-007 Kraków, Poland; magdalena.kostkiewicz@uj.edu.pl; 6 Nuclear Medicine Department, John Paul II Hospital, 31-202 Kraków, Poland; eborkowska@interia.com; 7 Department of Mass Spectrometry, Institute of Nuclear Physics, Polish Academy of Sciences, 31-342 Kraków, Poland; kamil.brudecki@ifj.edu.pl

**Keywords:** ^131^I, radioiodine, ^99m^Tc, technetium-99, occupational radiation exposure, nuclear medicine, human peripheral blood lymphocytes, confounding factors, physical and biological dosimetry

## Abstract

Nuclear medicine staff are constantly exposed to low doses of ionizing radiation. This study investigated the level of genotoxic effects in hospital employees exposed to routinely used ^131^I and ^99m^Tc in comparison with a control group. The study compared the results of physical and biological monitoring in peripheral blood lymphocytes. The effects of confounding factors, such as smoking status and physical activity, were also considered. Physical dosimetry monitoring revealed differences in the individual annual effective dose as measured by finger ring dosimeter and whole-body dosimeter between the ^131^I- and ^99m^Tc-exposed groups. The DNA damage studies revealed differences between the groups in terms of excess premature chromosome condensation (PCC) fragments and tail DNA. Physical activity and smoking status differentiated the investigated groups. When assessed by the level of physical activity, the highest mean values of tail DNA were observed for the ^99m^Tc group. When assessed by work-related physical effort, excess PCC fragments were significantly higher in the ^131^I group than in the control group. In the investigated groups, the tail DNA values were significantly different between non-smokers and past or current smokers, but excess PCC fragments did not significantly differ by smoking status. It is important to measure exposure to low doses of ionizing radiation and assess the potential risk from this exposure. Such investigations support the need to continue epidemiological and experimental studies to improve our understanding of the mechanisms of the health effects of radionuclides and to develop predictive models of the behavior of these complex systems in response to low-dose radiation.

## 1. Introduction

In recent years, the production and application of radioisotopes for diagnostic and therapeutic purposes in nuclear medicine have increased substantially [1]. The observed trend can be explained by developments in radioisotope properties and in imaging technology, which provides valuable data for patient decision making [2]. The most applicable advantages of radioisotopes are their biological half-life (in terms of human activity and metabolism), the ease of administration, and the low cost of isotope production [3,4]. Iodine-131 (also known as ^131^I, 131I, and radioiodine) and technetium-99 (^99m^Tc or 99mTc) are the most frequently used radioactive isotopes in nuclear medicine [4,5]. Due to its relatively short half-life (8.03 days) and selective uptake by the thyroid gland, radioiodine is used to diagnose and treat thyroid diseases [6,7]. Technetium is a metastable nuclear isomer of technetium-99 (half-life of 6.01 h) and is commonly used in ventilation-perfusion lung scintigraphy with the single-photon emission computed tomography (SPECT) imaging method [8].

Employees of nuclear medicine units are regularly exposed to various isotopes during their preparation and application to patients in the form of radiopharmaceuticals [9,10]. Nuclear medicine staff can be in close contact with patients during the diagnostic and therapeutic procedures [10,11]; therefore, radioactivity might be internalized via breathing air exhaled by the treated patient [7,8]. On this basis, it has been postulated that nuclear medicine personnel represent a group that is systematically exposed to low doses of ionizing radiation [12]. The actual dose received by personnel may depend on many factors, including the effective half-life, distance from the patient, administered activity, and time spent in proximity to the radionuclides and patients [7,8,12]. Many studies have shown that the effective dose for most diagnostic nuclear procedures is considered to be low (5–100 mGy) [12,13]. The current risk estimates are based on the linear no-threshold (LNT) risk model recommended by the International Commission of Radiological Protection and the Biological Effects of Ionizing Radiation VII report of the US National Academy of Sciences [14,15]. However, according to the LNT model, the lowest dose of radiation may still present a human health risk [16].

Our previous measurements of ^99m^Tc activity by a mobile aerosol sampler with a Petryanov filter in indoor air at the Nuclear Medicine Department (NMD), John Paul II Hospital, Kraków, Poland, during ventilation-perfusion lung scan treatments revealed annual effective doses of 1.6 µSv for technicians and 1 µSv for nurses [8]. However, the study concerned only one typical working day and only measured ^99m^Tc activity in the air; nevertheless, the estimated ^99m^Tc intakes were quite high [8]. Similarly, in the group exposed to ^131^I, we found that physical dosimetry may be insufficient to monitor the dose absorbed by the nuclear medicine staff [7]. Radioisotopes are taken up not only by target cells but also by human peripheral blood lymphocytes (HPBLs), which are known to be highly radiosensitive [7]. Thus, HPBLs are well-recognized as a suitable biological dosimetry model in studies of radiation exposure effects [12].

Currently, in Polish nuclear medicine units, workers’ exposure to external radiation is monitored only by thermoluminescent dosimeters (TLDs) [7,8,10]. However, TLDs do not provide any information on the dose absorbed by cells resulting from radioisotope exposure. ^131^I decays by the emission of β (e-) (806 keV) and γ (principally 364 keV) radiation [17]. During decay, Tc-99m emits gamma energy of 140.5 keV [8,18]. These types of radiation can induce various biological effects, such as different DNA damage profiles, changes in cellular chemical structures, and cell death [7,12]. One of the most harmful biological effects in cells resulting from ionizing radiation exposure is DNA damage, primarily double-strand breaks [7,12]. Because low radiation toxicity has mostly been estimated by extrapolating from observations at high doses, correlations between the doses absorbed by nuclear medicine staff and biological effects have been difficult to establish [15]. Furthermore, age, gender, exposure dose, working period, smoking status, physical activity, and nutritional habits could be confounders because they might modify the cellular responses to radiation exposure [7,19].

Studies on the genotoxic effects of low-dose occupational exposure in nuclear medicine units are mostly limited to physical monitoring or epidemiological observations [20,21], and the available data are inconsistent [12]. There is a lack of complex monitoring studies investigating how ^131^I and ^99m^Tc exposure impact cellular systems in nuclear medicine staff and how these biological effects are impacted by confounding factors. Systematic dose estimation of nuclear medicine staff constitutes an important element by which radioprotection organizations evaluate radiation risk and prepare protective protocols. Here, we evaluated genotoxicity in two groups of workers occupationally exposed to ^131^I or ^99m^Tc and compared the results with a non-exposed population. Complex physical and biological monitoring by TLD and biomarkers of DNA damage were applied in this study. A well-developed approach using a battery of methods, such as the cytokinesis-block micronucleus (CBMN) assay, premature chromosome condensation (PCC) assay, and comet assay in HPBLs to evaluate the genotoxic effects of the absorbed doses of ionizing radiation, was applied [7]. These are reliable and internationally accepted assays for detecting DNA damage and the cell division status. The influence of confounding factors, particularly the effects of smoking and physical activity, on DNA damage was also investigated.

## 2. Materials and Methods

### 2.1. Characteristics of the Study Population

The ^99m^Tc-exposed group contained nine employees (two men and seven women) who work with this radionuclide at the NMD, John Paul II Hospital in Kraków, Poland. The medical staff consisted of four technicians, three nurses, and two physicians. The detailed characteristics of the exposure group are presented in Table 1. The NMD specializes in SPECT diagnostics and has a Siemens Symbia T16 SPECT/CT hybrid device and a Technegas generator manufactured by Cyclomedica. Approximately 3600 diagnostic tests are performed annually, including heart scintigraphy (2000), kidney scintigraphy (200), bone scintigraphy (1000), and lung ventilation and perfusion scintigraphy (400) [8,22]. Technetium-99 was the only radiopharmaceutical in regular use during the monitored period. SPECT diagnostics were performed once per week (Tuesdays), usually for four patients per day [8,22]. In the first part of the SPECT examination, albumin containing 150 MBq of 99mTc is intravenously injected [8,22]. In the second step, a gas containing 400–500 MBq of ^99m^Tc is inhaled [8,22]. During each lung ventilation and perfusion scintigraphy test, a technician and a nurse are present. Details of the medical staff duties during the diagnostic procedures and descriptions of how the ^99m^Tc air activity and internal exposure were measured are given in our previous publications [8,22].

The iodine-exposed group consisted of 29 employees (2 men and 27 women) working in the Department of Endocrinology and Nuclear Medicine (DENM), Holy Cross Cancer Center, Kielce, Poland. In the DENM, ^131^I is administered to perform thyroid scintigraphy and to treat hyperthyroidism and thyroid cancer. The characteristics of the ^131^I group are presented in Table 1 of a previous publication [7]. The control group (1 man and 31 women) that was not exposed to radiopharmaceuticals was recruited from the administrative wards of the DENM [7]. Each participant was asked to complete a survey questionnaire, including personal data such as gender, age, occupational activity (also in the past), smoking status, dietary habits, physical activity, and previous radiation exposure. All participants were healthy at the time of blood sampling, and each was informed about the purpose and scope of the investigation. The participants signed a written informed consent form and a data protection statement. The peripheral blood samples from all participants were collected in heparinized vacutainers by phlebotomy at the beginning of a working day in the period between 2016 and 2020. The samples were immediately transported to the laboratory of the Department of Experimental Physics of Complex Systems, Institute of Nuclear Physics, Polish Academy of Sciences, Kraków, Poland, within 2 h at ambient temperature. The samples were de-identified and then immediately processed.

### 2.2. Iodine-131 and ^99m^Tc Blood Activity Measurements and Concentrations in Room Air

Blood activity measurements in the occupationally exposed groups were performed with a whole-body spectrometer a few weeks before blood collection [22,23]. Air activity measurements were also conducted [8,22,24]. More detailed information regarding the methodology of the measurements and the dose assessments can be found in previous publications [8,22,23,24].

### 2.3. Whole-Body Physical Dosimetry

As stated by the International Commission on Radiological Protection, the radiation dose to employees is expressed in terms of the effective dose and the equivalent dose for extremities and the eye lens [14]. As internationally recommended operational quantities for monitoring occupationally exposed persons, the Hp(10) value for the whole body and Hp(0.07) value for extremities were used [25]. The personal dose equivalent Hp(10) is the dose received by tissue (effective dose) at a 10 mm depth from the skin surface and reflects the dose delivered to the whole body [25]. The Hp(0.07) dose is the dose at a depth of 0.07 mm and reflects the dose received by the skin [25]. The external exposure dose received by the nuclear medicine staff was monitored using a whole-body passive thermoluminescence dosimeter (DI) and a finger extremity ring dosimeter (PI). Both dosimeters were provided by the Laboratory of Individual and Environmental Dosimetry, which was accredited to the EN-PN-ISO/IEC 17025 standard by the Polish Centre for Accreditation [25]. The dosimeters were changed every 4 months. The summarized doses in the ^99m^Tc-exposed group for every year between 2016 and 2020 are presented in Table 1. The data of the ^131^I-exposed group are presented in a previous publication [7].

### 2.4. Cytokinesis-Block Micronucleus Assay (CBMN)

The CBMN assay was performed as previously described [7]. All slides were coded and blinded to the scorer. The slides were analyzed for the micronuclei (MN) frequency and nuclear division index (NDI) according to the criteria and representative pictures provided by Fenech, M. [26]. The MN frequency was assessed based on an analysis of 1000 binucleated cells. The NDI was calculated for the first 500 cells. Decoding was conducted after completing the microscopic analysis of all slides from this study.

### 2.5. Premature Chromosome Condensation (PCC) Test

The cultures were treated similarly to those used in the CBMN assay according to a previously published protocol [7]. Instead of cytochalasin-B, calyculin A (50 nM) was added to the culture medium exactly 30 min before ending the culturing process (48 h). Cell finding and image capturing were performed on a Metafer4 scanning system equipped with a Zeiss Axio Imager Z2 microscope (MetaSystemsTM, Altlussheim, Germany). The PCC fragments per cell in excess of 46 PCC chromosomes for each donor were scored in 100 G2/M PCC-phase cells [7]. The representative micrographs presenting DNA damage in the PCC technique were presented in our previous article [27].

### 2.6. DNA Damage Measurement by Comet Assay

The standardized alkaline comet assay to evaluate the level of DNA damage was performed as described by Panek A. et al., with minor modifications [28]. For the molecular investigations, lymphocytes were isolated and carefully cryo-preserved in liquid nitrogen. Briefly, agarose-embedded cells were lysed for 1 h by 1% Triton X-100 detergent at an alkaline pH > 13. Next, the DNA was subjected to alkaline electrophoresis (30 min, 4 °C, 30 V, and 300 mA), which allowed for fragmented DNA migration. After ethidium bromide (17 mg/mL) staining, cellular DNA was visualized using an Olympus BX-50 epifluorescence microscope (Olympus, Tokyo, Japan). DNA damage was quantified using the comet T-DNA parameter (Komet 3.0, Kinetic Imaging Company, Liverpool, UK), where changes in the distribution of tail DNA are considered as a sensitive indicator of initial DNA breakage. Two independent experimental replicates were performed for each donor (2 slides, 100 cells from each slide). To check for possible instability in the experimental conditions, an internal standardization procedure was conducted as in a previous work [28].

### 2.7. Statistical Analysis

The data were analyzed using SPSS version 26 (SPSS Inc., Chicago, IL, USA) and Microsoft Office Excel 2013 for Windows. Figures were created using OriginPro 2020b (OriginLab, Northampton, MA, USA). The standard deviation (S.D.) was calculated for all measured biomarkers. Intergroup differences were assessed by the Kruskal–Wallis test followed by Dunn’s Pairwise post hoc comparison with a Benjamini–Hochberg correction [29]. *P* values of ≤ 0.05 were considered to be significant; the exact *p* values are given in the text.

## 3. Results

### 3.1. Internal and Physical Exposure Monitoring and Characteristics of the ^99m^tc Group

Table 1 presents the characteristics of the ^99m^Tc-exposed group, including subject code, age, work title, gender, duration of exposure, and internal exposure monitoring dose as measured by a whole-body dosimeter (DI) and a finger ring dosimeter (PI) for 2016–2020 for each nuclear medicine physician indicated by a TLD badge number.

In the ^99m^Tc group, the individual annual effective dose measured by DI ranged from 0.00 mSv to 1.89 mSv, with average values of 0.87, 0.58, 0.46, 0.45, and 0.05 mSv for the years 2016–2020, respectively (Table 1). All values were below the international recommended dose limit (20 mSv/year). By comparison, of the 29 individuals in the previously investigated ^131^I group [7], 24 had Hp(10) values lower than 0.4 mSv in 2016, with 26 in 2017. In 2016, three workers in the ^131^I group had Hp(10) values of 0.72, 2.31, and 0.42, with an average of 1.15 mSv [7]. In 2017, only one worker had an Hp(10) value higher than 0.4 [7]. As seen in Table 1, the highest annual dose in the 99mTc group recorded by PI was 116.56 mSv in 2017, with average values of 27.41, 32.95, 20.12, 16.22, and 5.90 mSv for the years 2016–2020, respectively. For ring dosimeters in the ^131^I group, two workers had values greater than 0.00, and the 2016–2017 average was 3.41 mSv. In the ^99m^Tc group, the DI and PI monitoring showed decreasing trends in the individual annual effective dose over the 2016–2020 time period.

### 3.2. Characteristics of the Examined Groups Stratified by Selected Confounding Factors

It is known that radiation alone may not be the only element affecting cellular responses, and radiation exposure might interact with other factors. Table 2 shows the characteristics of the three examined groups: the ^131^I group, the ^99m^Tc group, and the control group.

As presented in Table 2, the age and gender distributions were similar in the investigated groups. Among the groups, the ^99m^Tc group was older (with the highest age S.D.) and had the highest percentage of men (see Table 2). The workers enrolled in this study were classified into four groups based on their smoking status: current smoker, past regular smoker, past occasional smoker, and non-smoker. The percentage of past regular smokers and non-smokers was comparable between the groups; however, the percentage of current smokers and past occasional smokers differed among the groups.

Regarding physical activity, the data were analyzed as leisure time physical activity (LTPA) and work-related physical effort (WRPE) based on a self-report questionnaire. LTPA refers to physical activity during free time, i.e., recreational or household activity [30]. LTPA data was expressed as activity intensity measured by metabolic equivalents (METs) and duration (h/week) as MET-h/week. Regarding LTPA, each group was divided into two subgroups: normal activity (NA), indicating physical effort less than 25.04 MET-h/week (the cutoff represents the third quartile in the LTPA distribution), and high activity (HA), indicating physical effort greater than 25.04 MET-h/week. Comparable percentages of LTPA were observed in the ^131^I and ^99m^Tc groups for normal and high LTPA. However, in the control group, 74.1% of participants were classified as NA LTPA, and 25.9% were classified as HA LTPA (see Table 2). The WRPE data were also stratified into two groups: lower activity (LA), indicating < 100 MET-h/week, and HA, indicating ≥ 100 MET-h/week. By assessing the WRPE METs across the three groups, the highest disproportion was again seen for the control group (see Table 2). Only 25% of the control group had a WRPE of ≥100 MET-h/week.

### 3.3. Biological Monitoring via CBMN, PCC, and Comet Assays Confirmed Differences in Cell Response between Exposed and Control Groups

**Figure 1:**
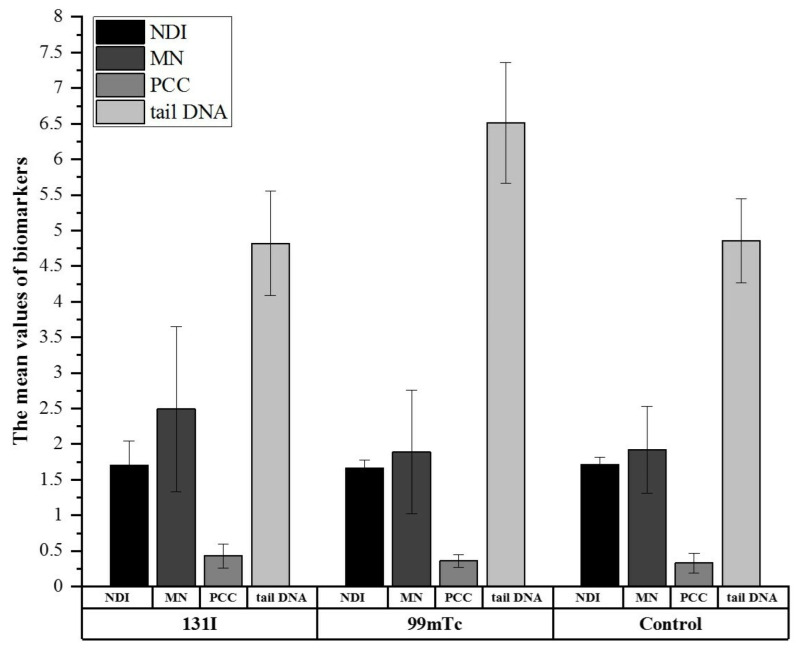
Mean values of biomarkers (NDI, MN, PCC, tail DNA) for human peripheral blood lymphocytes (HPBLs) of the ^131^I, ^99m^Tc, and control groups. The error bars represent the standard deviation. Tail DNA is DNA damage detected by a single-cell gel electrophoresis assay.

Cytogenetic and genotoxicity were assessed as the nuclear division index (NDI), micronuclei (MN) frequency, excess PCC fragments, and tail DNA in HPBLs. Figure 1 compares the mean biomarker values (i.e., NDI, MN, PCC, and tail DNA) among the groups.

The ^131^I group showed a significantly higher frequency of excess PCC fragments compared with controls (0.43 ± 0.17 vs. 0.33 ± 0.14; *p* ≤ 0.05). The ^99m^Tc group exhibited a significantly higher tail DNA value than the ^131^I group (6.51 ± 0.85 vs. 4.82 ± 0.73; *p* < 0.001). The tail DNA value was also significantly increased (*p* ≤ 0.001) in the ^99m^Tc group compared with the control group (6.51 ± 0.85 vs. 4.86 ± 0.59). The NDI and MN frequency were the highest in the ^131^I group; however, these biomarkers were not significantly different among the investigated groups (Figure 1).

### 3.4. Physical Activity and Smoking Status Differentiate Investigated Groups Based on DNA Damage

**Figure 2:**
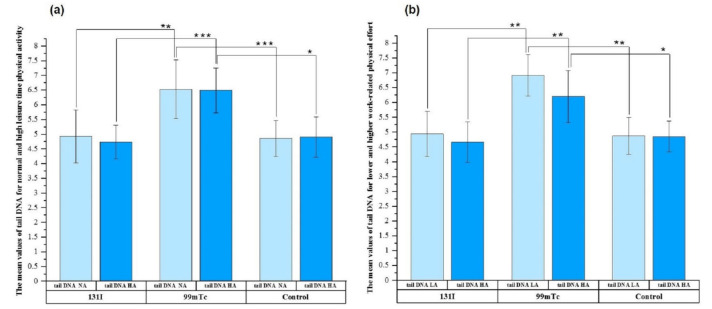
Mean values of tail DNA for normal (NA) and high (HA) leisure time physical activity (**a**), and low (LA) and high (HA) work-related physical effort (**b**) in the three studied groups. The error bars represent the standard deviation. * *p* ≤ 0.01; ** *p* ≤ 0.05; *** *p* ≤ 0.001.

To examine the contribution of NA and HA LTPA to DNA damage, plots for the exposed groups were created (Figure 2a). Analogous plots were also prepared for LA and HA WRPE (Figure 2b).

Generally, the results confirmed that the highest mean values of tail DNA for LTPA and WRPE were observed for the ^99m^Tc group. The mean tail DNA value for NA was significantly higher in the ^99m^Tc group compared with the ^131^I group (6.53 ± 1.00 vs. 4.93 ± 0.90, *p* ≤ 0.05) and control group (4.86 ± 0.61, *p* ≤ 0.001) (Figure 2a). An analogous trend was observed for the impact of HA LTPA on the tail DNA values (see Figure 2a).

The data presented in Figure 2b confirmed that both low and high (≥100 MET–h/week) WRPE influenced the tail DNA value. There were significant differences in LA WRPE between the ^99m^Tc group and both the ^131^I group (6.91 ± 0.70 vs. 4.94 ± 0.76, *p* ≤ 0.05) and control group (4.87 ± 0.62, *p* ≤ 0.05). Similar to the impact of HA LTPA on the tail DNA value, HA WRPE gave the highest tail DNA value in lymphocytes from individuals in the ^99m^Tc group (6.20 ± 0.88). Furthermore, there were significant differences between the ^99m^Tc group and the ^131^I group (*p* ≤ 0.05) and between the ^99m^Tc group and the control group (*p* ≤ 0.01).

**Figure 3:**
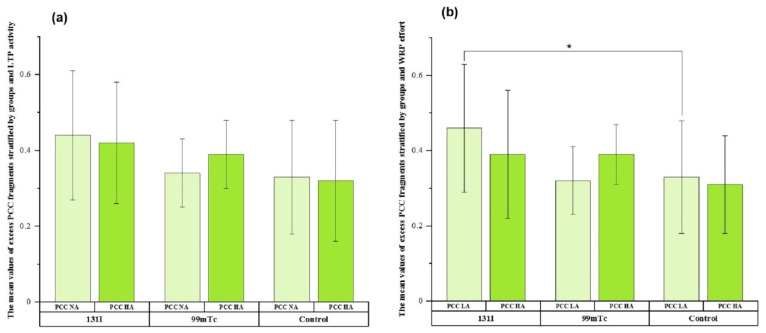
Frequencies of excess PCC fragments in NA and HA LTPA (**a**) and LA and HA WRPE (**b**) in peripheral lymphocytes of the three groups. * *p* ≤ 0.05.

Figure 3 shows the mean values of the excess PCC fragments detected for the investigated groups for NA and HA LTPA (Figure 3a) and NA and HA WRPE (Figure 3b).

For both NA and HA LTPA, the highest amount of excess PCC fragments was detected in the ^131^I group (Figure 3a). There was no significant difference in excess PCC fragments between NA and HA LTPA in any of the studied groups or between the three groups. By contrast, the excess PCC fragments for NA WRPE were significantly different between the ^131^I group and the control group (*p* ≤ 0.05). The differences within the studied groups between NA and HA WRPE were not significant.

**Figure 4:**
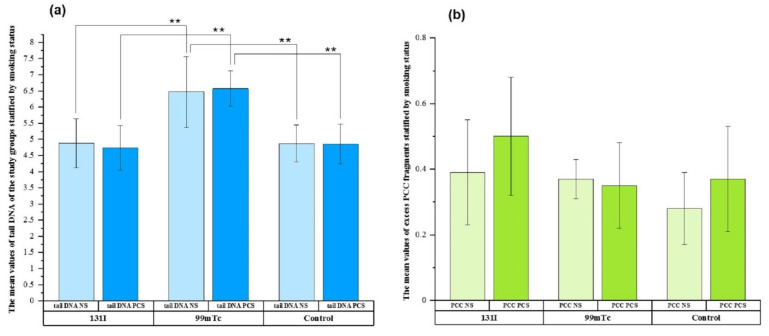
Tail DNA (**a**) and excess PCC fragments (**b**) stratified by smoking status (non-smokers (NS) and past or current smokers (PCS)) in the examined groups. ** *p* ≤ 0.05.

We also analyzed whether smoking status influenced DNA damage by examining tail DNA and excess PCC fragments. Figure 4a compares the mean tail DNA values in non-smokers (NS) and past or current smokers (PCS) by group. An analogous plot for excess PCC fragments is presented in Figure 4b.

The tail DNA values for both NS and PCS in the ^99m^Tc group (6.47 ± 1.09; 6.57 ± 0.55) were significantly higher than those in the ^131^I group (4.88 ± 0.76; 4.74 ± 0.69, *p* ≤ 0.05) and control group (4.87 ± 0.57; 4.86 ± 0.62), respectively (Figure 4a). Regarding excess PCC fragments and smoking status, no significant overall difference was found between exposed workers and controls. The highest value was found in the PCS in the ^131^I group, but it was not significantly higher than that of the other groups (Figure 4b).

## 4. Discussion

The purpose of this study was to systematically investigate the external exposure doses of ^131^I and ^99m^Tc and the genotoxicity associated with exposure to these radioisotopes in nuclear medicine staff. We also analyzed confounding factors to provide a more robust interpretation of the findings. Previous studies have shown that the radiological monitoring of nuclear medicine staff based on TLD measurements might not reflect real exposure [7] and is, therefore, insufficient. In the present study, we employed whole-body and partial-body physical monitoring by TLD, together with biological dosimetry methods to investigate peripheral blood lymphocytes. Data related to DNA damage were analyzed in terms of confounding factors, such as physical activity and smoking status.

Despite compliance with safety rules while working with radioisotopes, nuclear medicine personnel, such as nursing staff, physicists, technicians, and medical doctors, are exposed to ionizing radiation. Radiation exposure depends on many factors, such as the duration of exposure, distance, shielding, and work activities [31]. Previous studies have shown that ^99m^Tc- and ^131^I- exposed workers differ in their individual annual effective doses as measured by DI and PI [7]. Our DI and PI data confirmed that these groups differ in the amount of radiation exposure they receive while working in nuclear departments. In the ^99m^Tc group, the average dose was 0.48 mSv per worker per year; the highest observed dose was 1.89 mSv for donor 4 in 2016. In the ^131^I group, the average dose was 1.05 mSv per worker per year, and the highest individual dose was 2.31 mSv for donor 11 in 2016. In both groups, the highest radiation exposure values measured by both for DI and PI were observed for technicians and nurses. Notably, in the ^99m^Tc group, the DI- and PI- measured doses decreased over the 2016–2020 time period. The average PI-measured radiation dose was significantly lower in the ^131^I group (3.41 mSv) than in the ^99m^Tc group (20.52 mSv). However, we should be cautious in drawing strong conclusions because for some individuals in both groups there was no information about the Hp(10) and Hp(7) values. Nevertheless, these differences are important when discussing the average radiation exposure of workers in the studied groups and the rationality of using the two types of TLD badges.

Related to radiation exposure, an important factor in health assessment is radiation absorption by cells that are influenced by individual factors such the DNA damage repair process. External and internal exposure to radioisotopes can lead to cellular and DNA damage, not only in target cells but also in other cells, such as HPBLs [7]. Cytogenetic methods, such as CBMN and PCC assays using HPBLs, have been widely accepted as suitable for the biological monitoring of genetic damage in exposed populations in the workplace [7,32]. Notably, the mean age (46.9 ± 4.5 years for ^131^I vs. 51.3 ± 11.8 for ^99m^Tc and 48.0 ± 8.6 for controls) and gender distribution were similar among all investigated groups. Therefore, we did not analyze the potential impact of age and gender on the levels of DNA damage. However, our previous study investigated the impact of age and found that it impacted the cellular responses in the ^131^I group [7]. The average duration of exposure was also comparable (23 years for ^131^I vs. 21 for ^99m^Tc). The control group was matched for possible confounding factors. All of the individuals enrolled in the study were considered healthy; no participant disclosed an addiction to alcohol, and there were no special dietary preferences or restrictions.

Inspired by our previous observations, in the present study we evaluated the genotoxicity in a group of occupationally exposed workers and compared it with a non-exposed population using CBMN, PCC, and comet assays. Our biological monitoring studies confirmed that there were differences in the level of DNA damage between the exposed groups and the control group. The greatest differences were observed for tail DNA, as measured by comet assay (Figure 1). On average, lymphocytes of the ^99m^Tc group had significantly higher tail DNA values compared with the ^131^I and control groups. The mean tail DNA value of the control group correlated well with the range reported in the literature [33]. DNA damage is considered to be one of the primary serious consequences of exposure to ionizing radiation [34]. Even at low doses, radiation can induce several types of damage, such as oxidized bases, single-strand breaks (SSBs), and double-strand breaks (DSBs) [34]. Alkaline single-cell gel electrophoresis is known as the comet technique and is a widely accepted method for detecting SSBs, DSBs, and alkali-labile sites [28]. The presented data are consistent with other studies that have shown high levels of DNA damage in the peripheral lymphocytes of radiology technicians [19]. The highest average amount of DNA damage was observed in the ^99m^Tc group and was correlated with the TLD measurements, ^99m^Tc air activity, personnel blood measurements, and ventilation-perfusion SPECT lung scans [8,22]. The increased tail DNA in the ^99m^Tc group can be attributed to unrepaired SSBs or prolonged exposure to environmental clastogens in this group, which was not assessed by the PCC and CBMN methods. Some of the technicians and nurses from the ^99m^Tc group were equipped with personal aspirators, enabling air sampling to determine the radiation exposure at their workplaces [22]. For technicians, the maximum ^99m^Tc blood concentration levels reached 920 ± 70 Bq L-1 and 1300 ± 100 Bq L-1; for nurses, the maximum estimated activity concentrations were approximately 10 times lower [22]. Finally, the annual effective doses in ^99m^Tc blood were 34 µSv for technicians and 2 µSv for nurses [22]. For comparison, the estimated annual effective doses of ^131^I activity in the thyroids of nuclear medical staff ranged from 0.02–0.8 mSv [23].

Interestingly, in the present work, a significantly higher average frequency of excess PCC fragments was found only for the ^131^I group compared with the control group. The average values detected by the CBMN assay were higher in the exposed worker groups than in controls, but the NDI and MN frequency were not statistically different between the groups. Only a few studies have been conducted on radionuclide-induced DNA damage measured by cytogenetic methods, such as PCC [7,35]. Most of them were performed shortly after exposure. An ideal biological monitoring study should use a battery of tests to be sensitive to a wide dose range and radiation-specific changes. Therefore, in the present studies, we used different methods that are applicable to lower (CBMN and comet assays) and higher doses of radiation (PCC technique). Studies have shown that clinical doses of ^99m^Tc do not induce significant DNA lesions [16]. Therefore, the CBMN and PCC assays might not be sensitive enough to detect such lesions. In this regard, it should be noted that some people who are never exposed to radiation can still show spontaneous DNA lesions, which can arise from endogenous and environmental factors [7]. Spontaneous individual DNA lesions and their repair efficiency could have contributed to the observed differences in our study.

Although studies of radionuclide exposure have received much attention in recent years, some problems remain unresolved. There is still considerable debate regarding the biological impact not only of the radionuclides currently used in nuclear medicine but also of new therapeutics under development [36]. The available biophysical data postulate an LNT risk model for the effects of low-dose radiation. According to the LNT model, the lowest dose of ionizing radiation can have a stochastic effect and may be a potential risk for human health [15,37]. Some studies have postulated that chronic exposure to low-dose radiation can be not only genotoxic but also carcinogenetic [38]. Even though ^131^I and ^99m^Tc are short-lived isotopes, exposure to them might cause increased primary or secondary risk of cancer [39]. DNA damage after low-dose exposure can initiate danger signaling pathways and cell death [40]. A study by Zakeri F. et al. reported that doses of 8.14 ± 7.81 mSv/year for at least 5 years and below 50 mSv of cumulative dose induced subtle changes in priming the immune system [41]. The risks of cataracts and cardiovascular disease might also be increased by exposure to low doses of ionizing radiation [42]. For these reasons, establishing a safe dose for employee exposure is challenging.

We also assessed whether the level of DNA damage in exposed individuals was influenced by physical activity or smoking status. We found that the highest mean tail DNA values for both NA/HA LTPA and LA/HA WRPE were for workers occupationally exposed to ^99m^Tc. It has been suggested that a high level of LTPA is associated with decreased levels of DNA damage [43]. Our observations suggest that physical activity slightly decreased the levels of DNA damage in all three studied groups, but the differences were not significant. Significant differences were observed between the studied groups stratified by physical activity (see Figure 2a,b). This observation suggests that the type of exposure, rather than physical activity, differentiates the cellular responses to radiation. The PCC results showed a significant difference only for the NA WRPE between the ^131^I group and controls. The observed differences in DNA damage measured by comet assay and by the PCC method can be explained by the different types of genetic endpoints evaluated in the two techniques. Nevertheless, it should be highlighted that a donor’s age as well as sex may interfere with the response to low-dose radiation, resulting in different DNA damage profiles.

Regarding smoking status, there were significant differences in the tail DNA values between the NS and PCS groups. Once again, the highest values were observed for the ^99m^Tc group. No significant overall difference was found regarding excess PCC fragments. According to these results, smoking status seems to affect DNA damage, as detected by the comet assay, but further studies are needed in larger populations, especially for ^99m^Tc exposure. Concerning the effect of smoking status on DNA damage, data reported in epidemiological biomonitoring studies are contradictory [44,45]. Some studies have shown higher levels of cytogenetic biomarkers in smokers than in non-smokers. In contrast, another study found that smoking caused no significant elevation in chromosomal aberrations. In our study, we did not observe this phenomenon for any of the applied methods. This was probably because of the number of smokers in the study, which might have been too small to observe an effect of smoking on higher cytogenetic aberration frequencies.

Our main findings are supported by recent publications and provide evidence for the exposure of nuclear medicine personnel to radiation. However, this study has some limitations. A relatively small number of workers exposed to ^99m^Tc was included. Obtaining a larger sample size was difficult for a few reasons. Complex physical and cytogenetic monitoring studies are very time-consuming. Even if all exposed employees agreed to participate in the study, the nuclear medicine units in our region include no more than 10 people. Another reason for the small sample size was associated with the need for blood sampling in different regions and the implementation of COVID-19 restrictions, which significantly limited employee biomonitoring. An additional limitation is related to the use of dosimeters. There might have been unmonitored exposure due to taking off the dosimeter or working in different places. The occupational exposure of employees in nuclear medicine units is complex and differs over time between sites. Workers are exposed to various chemical substances used at work, which might be responsible for significant errors in biodosimetrical evaluations. The limitations of the applied tests must also be considered when interpreting the results. Studies with human peripheral lymphocytes are limited by many factors, such as the dose-detection limit of the applied method and the life span of the cells [7]. The observed disproportion in the numbers of donors, sex, and smoking status between the controls and the exposed groups can be considered a major limitation of the presented study. Nevertheless, these nonequivalences are frequently observed in human monitoring studies [19,31,42].

Even though the small sample size of the ^99m^Tc group precludes a definitive conclusion regarding increased DNA damage induced by occupational exposure, our experimental evidence nevertheless highlights the need for the continuous monitoring of exposed workers, especially those exposed to ^99m^Tc. The future biological consequences of occupational exposure are influenced by confounding factors and cannot be easily estimated by the currently available methods and models. This study is relevant in the context of radiation prevention programs and the health surveillance of radiological workers. It can be used to help establish monitoring systems and will promote work on discovering new low-dose measurement methods. The strengths of the present study are the investigation of different biological markers of radiation effects and discerning the susceptibility of different populations exposed to low levels of ionizing radiation by using TLD monitoring and whole-body spectrometry. In our opinion, this strategy should be applied in further worker monitoring studies to lay the foundation for strengthening radiological protection standards for employees who work with radionuclides.

## 5. Conclusions

Our results indicate that ^131^I- and ^99m^Tc-exposed groups differ in their occupational exposure to low-dose radiation in nuclear medicine settings. These differences were observed in the physical monitoring results and in the DNA damage measurements using peripheral blood lymphocytes. Confounding factors, such as physical activity and smoking status, differentiated the investigated groups in terms of their results from the applied biological methods. Although complex, this study highlights the need for epidemiological studies of low-dose radiation exposure in combination with experimental studies using various techniques. The long-term goal for this strategy is to improve our understanding of the mechanisms of the health effects of radionuclides and to improve biokinetic and predictive models of the behavior of these complex systems in response to low-dose radiation.

## Figures and Tables

**Table 1:** Characteristics of the ^99m^Tc-exposed group, including age, work title, gender, duration of exposure, and dose (mSv) measured by DI and PI for 2016–2020.

Subject Code	Age (y)	Profession	Gender	DoE (y)	DI	PI	DI	PI	DI	PI	DI	PI	DI	PI
					2016		2017		2018		2019		2020	
**1**	58	Nurse	F	15	1.60	7.35	1.08	1.80	1.28	1.98	0.97	2.29	0.00	0.53
**2**	30	Technician	F	8	1.10	68.51	0.84	116.56	0.45	46.81	0.83	65.04	0.06	21.99
**3**	68	Medical doctor	F	40	0.01	-	0.00	-	0.00	-	0.00	-	0.00	-
**4**	55	Technician	F	34	1.89	52.88	1.74	48.90	1.24	50.32	0.72	26.49	0.25	6.68
**5**	49	Medical doctor	M	24	0.08	-	0.05	-	0.00	-	0.00	-	0.00	-
**6**	55	Nurse	F	16	1.07	2.93	0.57	0.81	0.24	1.24	0.50	0.80	0.02	0.29
**7**	55	Technician	F	34	0.46	27.00	0.26	29.56	0.09	20.01	0.02	16.21	0.06	5.92
**8**	57	Nurse	F	19	0.73	5.81	0.67	0.04	0.82	0.36	1.04	0.44	0.07	0.00
**9**	35	Technician	M	4	-	-	0.01	-	0.00	-	0.00	2.30	0.00	-

DI—whole-body dosimeter, DoE—duration of exposure, F—female, M—male, PI—finger ring dosimeter, —no data.

**Table 2:** Characteristics of the examined groups with respect to mean age, sex, smoking status, leisure time physical activity, and work-related physical effort. The control group was used as a match for possible confounding factors that may affect cell responses.

Confounding Factors	^131^I	^99m^Tc	Controls
	[*n* = 29]	[*n* = 9]	[*n* = 32]
**Age**	46.9 ± 4.5	51.3 ± 11.8	48.0 ± 8.6
Mean (S.D.)			
**Sex (%)**	93.1	77.8	96.9
Women	6.9	22.2	3.1
Men			
**Smoking status (%)**	10.3	0	15.6
Current smoker	24.1	22.2	28.1
Past regular smoker	3.5	22.2	3.1
Past occasional smoker	62.1	55.6	53.1
Non-smoker			
**Leisure-time physical activity (%)**	53.8	55.6	74.1
Normal			
(<Q3: 25.04 MET-h/week)	46.2	44.4	25.9
High			
(>Q3: 25.04 MET-h/week)			
**Work-related physical effort (%)**	58.6	44.4	75
(<100 MET-h/week)	41.4	55.6	25
(≥100 MET-h/week)			

MET—metabolic equivalents, S.D.—standard deviation, Q—quartile.

## Data Availability

The data presented in this study are available on request from the corresponding author.
